# *Borrelia burgdorferi* Outer Membrane Vesicles Contain Antigenic Proteins, but Do Not Induce Cell Death in Human Cells

**DOI:** 10.3390/microorganisms10020212

**Published:** 2022-01-19

**Authors:** Kati Karvonen, Hanna Tammisto, Jonna Nykky, Leona Gilbert

**Affiliations:** 1 Department of Biological and Environmental Science and Nanoscience Center, University of Jyvaskyla, P.O. Box 35, FI-40014 Jyvaskyla, Finland; tammisto.hanna@gmail.com (H.T.); jonna.nykky@jyu.fi (J.N.); 2 Te?ted Oy, Mattilanniemi 6-8, FI-40100 Jyvaskyla, Finland

**Keywords:** Lyme borreliosis, bleb, extracellular vesicle, persistent antigen

## Abstract

Like many bacterial species, *Borrelia burgdorferi*, the pleomorphic bacterium that causes Lyme borreliosis, produces outer membrane vesicles (OMVs). Borrelial OMVs (BbOMVs) have been identified as containing virulence factors, such as outer surface proteins (Osps) A, B, and C, as well as DNA. However, the pathogenicity of BbOMVs in disease development is still unclear. In this study, we characterized purified BbOMVs by analyzing their size and immunolabeling for known antigenic markers: OspA, OspC, p39, and peptidoglycan. In addition, BbOMVs were cocultured with human non-immune cells for cytotoxicity analysis. The results demonstrated that, on average, the vesicles were small, ranging between 11 and 108 nm in diameter. In addition, both OspA and OspC, as well as Lyme arthritis markers p39 and peptidoglycan, were detected from BbOMVs. Furthermore, BbOMVs were cocultured with non-immune cells, which did not result in cell death. Combined, these results suggested that BbOMVs could participate in the induction of infection by functioning as a decoy for the host immune system. Furthermore, BbOMVs might serve as a means for persistent antigens to remain in the host for prolonged periods of time.

## 1. Introduction

Lyme borreliosis (LB), the most common vector-borne disease in North America and Europe [1], is caused by the pleomorphic spirochete bacteria *Borrelia burgdorferi* (*B. burgdorferi*) [2]. LB can present with mild flu-like symptoms with or without a red rash called erythema migrans; however, especially if not treated early, the infection can develop into a more severe multisystemic disorder with manifestations in the skin, joints, central nervous system, and/or heart [3,4]. Currently, the exact mechanisms resulting in prolonged sequelae of the disease are still unknown. However, persistence of the bacterium or bacterial antigens in the host tissue are considered as one possible explanation for continued disease manifestations [5,6,7,8,9,10].

Bacterial outer membrane vesicles (OMVs) are produced in normal growth cultures by Gram-negative bacteria [11]. Currently, it is believed that OMVs are produced either by blebbing of the bacterial outer membrane or explosive cell lysis [12]. OMVs are spherical, 10–300 nm in size, and contain a single membrane bilayer [11,13]. Furthermore, OMVs consist of similar outer membrane proteins, polysaccharides, and lipids as the membranes of the originating bacterium, and can carry a variety of cargo, including genetic information [11,12,14]. Moreover, OMVs have been identified from in vitro growth cultures, as well as in vivo animal and human fluid and tissue samples [15,16,17,18,19,20]. Hence, it has been suggested that OMVs function, for example, as a means for the bacterium to react to the surrounding environment, or in inter- and intracellular communication, the transport of biological signals far away from the originating bacterium, the removal of harmful factors from the surface of the bacterial cell, or as decoy targets for host immune response and transforming agents, among other things [11,12,14,21,22]. 

*B. burgdorferi* OMVs (BbOMVs), or blebs, have been purified from naturally producing in vitro cell cultures [23,24] and by chemically inducing the formation of blebs in *Borrelia* cultures [25]. Furthermore, BbOMVs have been visualized from in vivo samples [16]. BbOMVs have been revealed to contain outer surface proteins (Osps) A, B, C, and D, as well as several unidentified proteins with sizes of 14–110 kDa [16,24,26,27,28], but lack flagellar proteins [16,24,26]. Both linear and circular DNA, from mostly plasmid origin [23,29], as well as plasmid-encoded RNA transcripts, are contained in BbOMVs [30]. In Table 1 below, some of the known characteristics of BbOMVs are listed. Furthermore, BbOMVs have been demonstrated to attach to human umbilical vein endothelial cells [24], as well as bind and enter human fibroblast, dendritic, T, and B cells [31], thus proving the ability of these vesicles to interact with host cells.

Borrelial whole-cell antigens, as well as peptidoglycan, RNA, and DNA have been detected from tissue samples of both animal and human patients after receiving antibiotic treatment [7,8,9,10]. BbOMVs could provide a locus for these antigens, thus affording an explanation for antigenic persistence without the discovery of viable spirochetes from antibiotic treated samples. In this study, the diameter of naturally formed BbOMVs was measured for the first time. Further characterization of BbOMVs by electron microscopic analysis and immunostaining methods was performed. Furthermore, the purified vesicles were cocultured with human non-immune cells for cytotoxicity analysis. The results indicated that BbOMVs were, on average, 33 nm in diameter, and that they contained known antigenic markers OspA, OspC, p39, and peptidoglycan. However, the vesicles did not induce cell death in chondrosarcoma or dermal fibroblast cells after 72 h of coculture.

## 2. Materials and Methods

### 2.1. Bacteria Cultures

Infectious *B. burgdorferi* strains B31 (ATCC, 35210) and GCB726 with fluorescent green protein (GCBC), kindly provided by Georges Chaconas [33], were utilized in the experiments. Barbour–Stoenner–Kelly (BSK II) medium [34], without gelatin and supplemented with 6% heat inactivated rabbit serum (Sigma, St. Louis, MO, USA), was used to grow both bacteria at immunologically relevant +37 °C. Low passage number (p8 or less) bacterial cells were used in all experiments. In caspase activation analysis (Section 2.6), round body forms (RBs), used as a control, were induced as previously described [35]. Before each experiment, *B. burgdorferi* cells were counted with a C-Chip DHC-N01 Disposable Haemocytometer (System Neubauer Improved; Digital Bio, Washington, DC, USA).

### 2.2. Human Cell Cultures

Chondrosarcoma (SW1353, HTB-94) and normal dermal fibroblast (BJ, CRL-2522) cell lines were acquired from American Type Culture Collection. SW1353 and BJ cell lines were utilized for their relevance as a disease-related model for arthritis and skin manifestations, respectively [36,37]. SW1353 cells were grown in Leibovitz’s L-15 media (Sigma), supplemented with 10% fetal bovine serum (Gibco, Paisley, UK), 2 mM l-glutamine (Gibco), and 100 IU/mL Penicillin/0.2 mg/mL streptomycin (Gibco) antibiotic cocktail, and incubated at +37 °C with 100% air. BJ cells were grown in Eagle’s minimum essential media (Sigma), with the above-mentioned supplements and an additional 1 mM sodium pyruvate (Gibco), and incubated at +37 °C, 5% CO_2_.

### 2.3. BbOMV Purification

BbOMVs were purified as previously described with modifications [24,38]. First, BbOMVs were produced by culturing 100 million *B. burgdorferi* cells in 25 mL of BSK II media to log phased (four days) growth. The bacterial cells were removed by centrifugation at 1000× *g* for 30 min (Thermo Scientific SL 16R Centrifuge, Waltham, MA, USA). By using 0.2 µm filters (Filtropur S plus 0.2, Sarstedt, Numbrecht, Germany) and syringe gravity filtering, the supernatant was further cleared from any bacterial remains. A Leica DM5500 fluorescence microscope with 20× objective, was utilized to confirm the lack of bacteria in the supernatants. Next, the samples were concentrated with 100 kDa Amicon Ultra-15 centrifugal filter unit (Merck, Kenilworth, NJ, USA) spinning at 3000× *g* for 15 min at RT, before ultracentrifugation at 100,000× *g* for 120 min at +4 °C (Beckman Coulter Optima L90-K Ultracentrifuge, 70 Ti-rotor, Brea, CA, USA). The formed BbOMV pellets were resuspended into 50–100 µL of cold 5 mM MgCl_2_ in PBS, and the protein concentrations were measured using NanoDrop. BbOMVs were stored at +4 °C before being cocultured with human cells within 7 days, and, for SDS-PAGE, stored at −20 °C. Negative stained transmission electron microscopy (TEM) samples were prepared immediately after purification.

### 2.4. Transmission Electron Microscopy

TEM was utilized in visualizing negatively stained freshly purified and epon embedded BbOMVs. A 10 µL drop of freshly purified BbOMVs, with a protein concentration of 20 mg/mL, was placed on glow discharged (EMS/SC7620 Mini Sputter Coater, Hatfield, PA, USA) grid for 20 s before negative staining with 1% phosphotungstic acid for 30 s. For better visualization of the double membrane of BbOMVs both BbOMV and control sample of *B. burgdorferi* spirochetes were prepared for epon embedded thin sections as previously described [39]. In brief, after BbOMV purification several pellets were combined by centrifugation with Airfuge centrifuge (Beckman Coulter, Brea, CA, USA, A-95 rotor, 22 psi, 30 min) and fixed with 2% glutaraldehyde in 0.1 M phosphate buffer for 10 min. For the control sample, the bacterial cell pellet from the vesicle purification was washed twice with PBS and fixed as above. Both samples were pelleted in a swing-out rotor at 2700× *g* for 10 min RT (Heraus Megafuge 1.0 R, Hanau, Germany). 1% uranyl acetate was used to stain epon embedded thin sections as previously described [39]. JEOL JEM1400 transmission electron microscope was utilized in imaging all the samples.

During imaging, residual *Borrelia* structures from the purified BbOMV samples was detected. Hence, the ratio of the residual *Borrelia* and BbOMVs was analyzed by counting BbOMVs and *B. burgdorferi* cells from 30 randomly selected images with 20,000× magnification and an area of 5.04 mm^2^. Particles with a heterogeneous but rounded shape, sized < 200 nm, with a visible membrane, even if partial, and light or dense inside, were considered as BbOMVs. On the other hand, particles with round or spherical shapes, sized > 200 nm, and visible membrane layer enclosing a dense structure were determined as borrelial residues.

### 2.5. Characterization of BbOMVs

#### 2.5.1. BbOMV Size Analysis

The size of purified BbOMVs was determined from TEM images of freshly-purified, negatively-stained, and epon-embedded BbOMV samples using ImageJ [40]. After brightness and contrast adjustments, a binary image was formed, from which the number of particles and their surface areas were automatically calculated, and the diameter of each vesicle was determined. The sizes were calculated from three separate BbOMV purifications, with a total of 600 BbOMVs analyzed.

#### 2.5.2. Qubit Analysis

The presence of DNA in the purified BbOMVs was quantified with a Qubit 2.0 fluorometer (Life Technologies, Carlsbad, CA, USA). *B. burgdorferi* cell lysates, from both GCBC and B31 strains, were utilized as positive controls, while albumin was a negative control, and BbOMVs from both borrelial strains were analyzed. A 1 mg/mL protein concentration stock solution from each sample was prepared. Using the broad range assay kit (Qubit dsDNA BR assay kit, 2–1000 ng range, Invitrogen, Eugene, OR, USA) and a sample volume of 5 µL, in 195 µL of Qubit working solution, the samples were analyzed as instructed by the manufacturer. Standard deviations from three replications are presented.

#### 2.5.3. SDS-PAGE

To resolve proteins in BbOMV samples, 12% SDS-PAGE gels were used. The protein concentrations were determined with a NanoDrop One spectrophotometer (Thermo Scientific, Madison, WI, USA) and 20 µg per sample were analyzed. A pre-stained ladder (precision plus protein kaleidoscope pre-stained protein standards, 10–250 kDa, BioRad, Hercules, CA, USA) was used. Albumin (Sigma) was utilized as a positive control for Coomassie staining. Borrelial cell lysates from GCBC and B31 strains, positive controls for borrelial proteins, were lysed by boiling and sonicating *Borrelia* cell pellets at +95 °C for 15 min each. Water was used as a negative control for staining. Both GCBC and B31 *B. burgdorferi* purified BbOMVs were examined. All samples were prepared in Laemmli buffer and boiled twice, for 5 min at +95 °C, before running the gels in 200 V for 30–45 min. Coomassie Brilliant Blue (10–15 min, shaking) and a de-staining solution (10% acetic acid, 20% MeOH) were utilized in visualizing protein bands.

For glycoprotein detection, a Pierce Glycoprotein staining kit (Thermo Fisher Scientific, Rockford, IL, USA) was used, according to manufacturer’s instructions. A total of 60 µg of protein for each sample, except for positive and negative controls (provided by the kit), which had 20 µg of protein each, as instructed by the manufacturer, was utilized. Horseradish peroxidase and soybean trypsin inhibitor were the positive and negative controls, respectively, provided by the kit.

Both Coomassie- and glycoprotein-stained gels were imaged with ChemiDoc MP (Bio-Rad, Hercules, CA, USA), and the images further quantified by each lane with the ImageJ gel analyzer tool. The experiments were performed three times.

#### 2.5.4. Western Blot

Western blotting and immunolabeling were utilized in further characterizing the BbOMVs. Proteins were separated by SDS-PAGE, similar to the protocol mentioned above in Section 2.5.3. *B. burgdorferi* cell lysates, from both GCBC and B31 strains, were utilized as positive controls, while albumin was a negative control, for the labels. A total of 100 µg of protein for the BbOMV samples was used. Similarly, for OspC, p39, and peptidoglycan labels, the amount of *B. burgdorferi* cell lysates was 100 µg, while 5 µg was utilized for OspA and *B. burgdorferi* labels. A total of 50 µg of albumin was used in each blot. The proteins were transferred onto nitrocellulose membranes (Protran BA 83, GE Healthcare, Chalfont Saint Giles, UK) by blotting at 100 V for 60 min. The membranes were blocked for unspecific binding with 3% bovine serum albumin (BSA) in TBS, for at least 30 min at RT, or overnight at +4 °C. Immunostaining was performed with the following five primary antibodies: mouse anti-*Borrelia burgdorferi* OspA (Santa Cruz Biotechnology, Dallas, TX, USA, sc-58093), mouse anti-peptidoglycan clone 3F6B3 (Bio Rad, 7263-1006), rabbit anti-p39 (Rockland Antibodies and Assays, 200-401-C17S), rabbit anti-OspC (antibodies-online.com, ABIN964717), and rabbit anti-*Borrelia burgdorferi* (Bio Rad, 1439-9406). The primary antibodies were diluted into 3% BSA, 0.2% Tween20-TBS at a 1:1000 dilution, except for the anti-peptidoglycan, which was used at a 1:200 dilution. The blots were incubated with the primary antibodies for 1 h at RT, while shaken. Four washes followed, 5 min each with washing buffer (0.2% Tween20-TBS), after which the secondary antibodies were incubated for 30 min at RT, while shaken. The secondary antibodies were rabbit anti-mouse (D0306) and swine anti-rabbit (D0314) AP-conjugated antibodies (Agilent Dako, Glostrup, Denmark), and both were used at a 1:500 dilution in 3% BSA, 0.2% Tween20-TBS. Another five washes with washing buffer preceded the equilibration of the membranes in APA buffer (0.1 M tris/HCl, 0.1 M NaCl, 5 mM MgCl_2_) for 5–10 min, followed by the colorimetric solution (APA buffer, Nitro Blue Tetrazolium (330 µg/mL), 5-bromo-4-chloro-3-indolyl phosphate (165 µg/mL)), which was stopped using dH_2_O.

The membranes were imaged with ChemiDoc MP. The specific bands for OspA, OspC, and p39, and the whole lanes for peptidoglycan and *B. burgdorferi* labeled blots were further quantified with the gel analyzer tool in ImageJ. The densitometry values were normalized to the GCBC lysate, except the B31 BbOMV, which was normalized to the B31 lysate. The amount of residual *Borrelia* was taken into consideration by subtracting the percentage value from the analyzed signal intensities of BbOMVs. The experiments were repeated three times.

### 2.6. Caspase Activation Analysis

In order to investigate the cytotoxic effect of BbOMVs on human cells, flow cytometry analysis of cell viability was performed. The cells were infected as previously described [41]. Briefly, 30,000 SW1353 and BJ cells were seeded onto 24-well plates and allowed to attach overnight. The plates were washed with +37 °C PBS before the addition of *B. burgdorferi* spirochetes and RBs (MOIs 40), as well as 50 µg/mL of BbOMVs. The plates were incubated on ice for 1 h in order to synchronize cell entry, after which each cell line’s antibiotic-free media was added to the wells, and the plates were incubated for 72 h at +37 °C. Untreated cells were used as negative control, while 2 h incubation with 1 µM staurosporine (S4400 staurosporine from *Streptomyces* sp., Sigma) was a positive control for apoptosis. After 72 h, the samples were washed twice with +37 °C PBS and trypsinized by placing 150 µL of 0.05% trypsin/EDTA (Sigma) into each well and incubating the plates at +37 °C for 5 min. Once the cells detached from the wells, the activity of trypsin was stopped by the addition of each cell line’s media. Guava MultiCaspase FAM kit (4500-0530, Merck, Darmstadt, Germany) utilizes a pan-caspase inhibitor (VAD), which is conjugated to carboxyfluorescein (FAM) fluorochrome and a fluoromethyl ketone group (FMK), which covalently binds the inhibitor to an activated caspase. Furthermore, the kit includes a DNA dye (7-AAD) as an indicator of membrane integrity. Hence, the FAM-VAD-FMK pan-caspase inhibitor, together with the 7-AAD DNA dye, was used to analyze the stage of apoptosis initiated by caspases in the human cells, according to the manufacturer’s instructions. The samples were analyzed in round-bottomed 96-well plates (Corning, Corning, NY, USA) with a Guava^®^ easyCyte 8HT benchtop flow cytometer (Millipore, Burlington, MA, USA), using the following settings: blue and red lasers on, 5-decade acquisition, threshold for forward scatter at 3000, 525/30, and 695/50 nm filters, with 10.6% compensation in the red channel for green signal. The appropriate settings were adjusted using unstained, as well as separately stained, negative and positive control samples. Either 10,000 events or 3 min acquisition time was utilized in acquiring the data in the Guava InCyte 3.0 software, which was also used for data analysis. The viable, early apoptotic, late apoptotic/necrotic, and dead cell populations were distinguished using quadrant regions in the dot plots. Each experiment was performed three times with triplicate samples.

### 2.7. Statistical Analysis

Statistical analysis was performed for the Caspase activation analysis samples (Section 2.6). A two-tailed, unequal variance student’s *t*-test was utilized to compare both the untreated and staurosporine treated controls to the *B. burgdorferi* samples. Significance was assumed for samples with the following levels: * *p* ≤ 0.05; ** *p* ≤ 0.01; *** *p* ≤ 0.001. Microsoft excel was employed in the analysis.

## 3. Results

### 3.1. BbOMVs Were on Average 33 nm in Diameter

**Figure 1:**
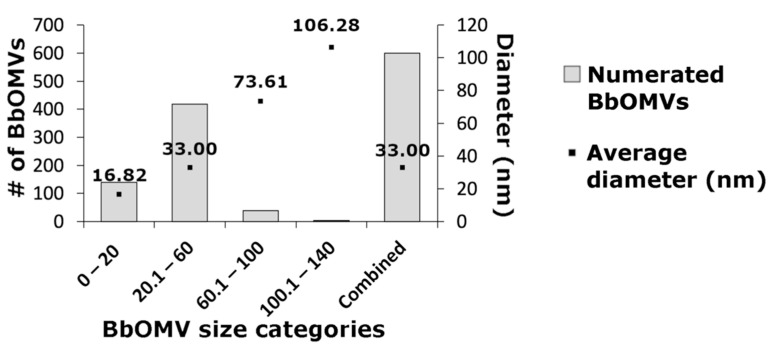
*B. burgdorferi* outer membrane vesicles (BbOMVs) were, on average, 33 nm in diameter. Purified BbOMVs were numerated (n = 600) and their diameters measured from negatively stained transmission electron micrographs, and were then divided into four size categories: 0–20, 20.1–60, 60.1–100, and 100.1–140 nm. The average diameters in each category are presented. The combined average diameter of a BbOMV was 33 nm.

Ultracentrifugation with filtering and concentration steps was utilized in purifying BbOMVs from log phased *B. burgdorferi* cultures. The purified vesicles were measured from negatively stained TEM micrographs, and their diameters calculated from the widest section of the vesicles (Figure 1). In total, 600 BbOMVs were analyzed with ImageJ, and, based on the diameters, four size categories were established: 0–20 nm, 20.1–60 nm, 60.1–100 nm, and 100.1–140 nm (Figure 1). Most of the analyzed BbOMVs belonged to the 20.1–60 nm category, which contained over 418 vesicles. Only three analyzed vesicles were over 100 nm in diameter (Figure 1). The smallest diameter was 11.41 nm and the largest 107.70 nm, while the average was 33.00 nm. Often, the smaller (<60 nm) vesicles were in clusters while larger ones were found separately.

**Figure 2:**
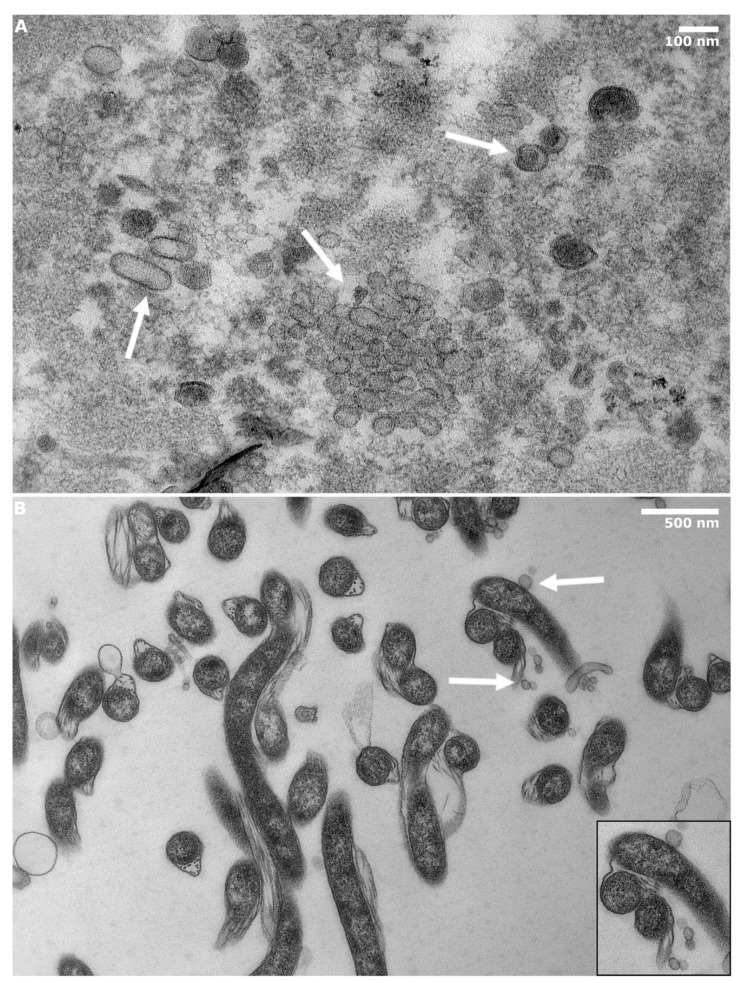
BbOMVs could be successfully and reproducibly purified from *Borrelia* cultures. (**A**) Transmission electron micrographs of epon embedded samples of purified BbOMVs demonstrating spherical shapes with single bilayer membranes. (**B**) As a control for purification, epon embedded *B. burgdorferi* spirochetes with a zoomed image (black box) of blebbing and BbOMVs directly originating from the bacterial cells are presented. White arrows indicate both clusters and separate BbOMVs in the purified vesicle and spirochete samples. Scale bars: A: 100 nm, B: 500 nm.

In Figure 2A, BbOMVs of heterogenic, but mostly rounded, shapes with a single membrane bilayer can be observed. Both light and dense BbOMVs were detected. The difference in opacity of the vesicles was most likely due to ruptured membranes, which allowed the stain to leak inside the vesicles (Figure 2A). A control image of *B. burgdorferi* spirochetes with vesicles blebbing off can be distinguished in Figure 2B (white arrows and zoomed image).

From epon-embedded BbOMV micrographs, residual *B. burgdorferi* were located (Figure S1). Therefore, the ratio between BbOMVs and residual *Borrelia* was calculated from 30 randomly selected images, where only 1.8% of borrelial spirochetes were detected. Hence, the purification protocol was still considered successful, and the characterization and further utilization of purified BbOMVs was valid.

### 3.2. Known Antigenic Markers Were Located in BbOMVs

**Figure 3:**
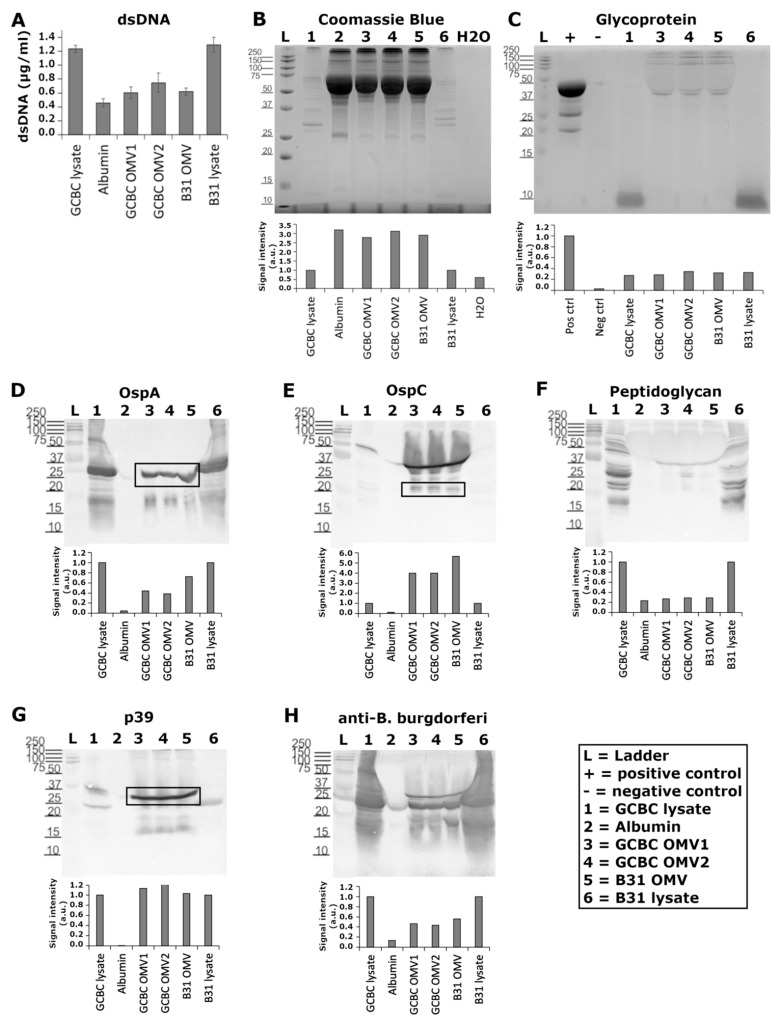
*B. burgdorferi* outer membrane vesicles contained several antigenic markers. (**A**) BbOMVs contained double stranded DNA, which was measured using a Qubit 2.0 fluorometer with a broad-range stain. Standard deviations from triplicate experiments. (**B**) Proteins were resolved with SDS-PAGE and stained with Coomassie Blue, while a Pierce Glycoprotein staining kit (Thermo scientific) was used for visualizing glycoproteins (**C**). Western blot analysis demonstrated known borrelial antigens OspA (**D**), OspC (**E**), peptidoglycan (**F**), and p39 (**G**). Anti-*B. burgdorferi* whole cell antibody was examined as a control (**H**). For the protein analysis, H_2_O was used as a negative control, and albumin (lane 2) as a positive control, for the staining. The negative (−, soybean trypsin inhibitor) and positive (+, horseradish peroxidase) controls provided by the glycoprotein staining kit were employed in the analysis. In the Western blots, albumin was utilized as a negative control for the immunolabel. *B. burgdorferi* strains GCBC (lane 1) and B31 (lane 6) bacterial cell lysates were used as positive controls for the labels in each experiment. Lanes 3 and 4 demonstrate BbOMVs purified from CGBC culture, while lane 5 has B31 purified BbOMVs. In order to obtain the full range of signals, the intensity values were analyzed from each lane in the Coomassie Blue and glycoprotein gels, as well as in the peptidoglycan and anti-*B. burgdorferi* labeled blots. Whereas the signals from specific bands representing each expected protein (black box) in OspA, OspC, and p39 blots were examined. Values were normalized to the positive controls: borrelial cell lysates in Coomassie Blue stained gel and western blots, and the kit provided positive control (horseradish peroxidase) in the glycoprotein gel. GCBC BbOMVs and albumin were normalized to GCBC lysates, and the B31 BbOMVs to the B31 lysate. Representative images from three separate experiments.

Purified BbOMVs were further characterized for the presence of double-stranded DNA, as well as a variety of proteins and glycoproteins (Figure 3A–C). By utilizing a Qubit assay kit and fluorometer, dsDNA could be easily detected from the purified BbOMVs (Figure 3A, lanes 3–5). Although some background was evident as demonstrated by the signal in the negative control (albumin, lane 2), there was still more signal in the BbOMV samples, suggesting the presence of dsDNA inside the vesicles (Figure 3A, lanes 3–5).

In Figure 3B–H, the respective images of gels and blots of BbOMVs analyzed for proteins, glycoproteins, and specific protein immunolabels, respectively, are shown. Below each image, the respective densitometry analyses of the labels are reported. Firstly, proteins were visualized with Coomassie Blue stain, which demonstrated visible bands in each lane except the negative control (H_2_O) (Figure 3B). Both GCBC and B31 bacterial cell lysates (lanes 1 and 6) demonstrated visible protein bands ranging from 10–250 kDa in size (Figure 3B). The positive control for proteins, albumin (lane 2), exhibited several bands, although the most visible band was ~66 kDa in size, as expected (Figure 3B). However, BbOMVs (lanes 3–5) presented comparable bands, suggesting traces of albumin from the media in the purified vesicle samples. From the densitometry analysis below the gel, this trend can be observed, since albumin (lane 2) had the highest value, closely followed by the BbOMV samples (Figure 3B, lanes 3–5).

Secondly, glycoprotein bands in the BbOMV samples (lanes 3–5) were visible in the range of ~40–250 kDa, while in the lysate controls (lanes 1 and 6) only very small (below 10 kDa) bands were detected (Figure 3C). Moreover, the signal intensity analysis clearly demonstrated the existence of glycoproteins in each sample, as the lysates and BbOMVs had similar intensity values (Figure 3C).

Lastly, the western blot analysis of OspA, OspC, and p39 illustrated visible bands in BbOMV lanes at the expected sizes for each protein: 28, 20.7, and 39 kDa, respectively, as demonstrated by both the representative images and the densitometry analysis for each protein (Figure 3D,E,G, respectively). Curiously, OspC bands were almost undetectable in both bacterial cell lysate lanes, as visualized by the blot and the low signal intensity values in Figure 3E (lanes 1 and 6). Additionally, the lysate bands in the peptidoglycan blot demonstrated a variety of sizes, while BbOMVs illustrated a 37 kDa protein in each lane, and two smaller bands with sizes of 25 and ~26 kDa in one of the BbOMVs (lane 4) (Figure 3F). The signal intensities for BbOMVs (lanes 3–5) in the glycoprotein blot mirrored the blot image, as the signals were not as strong as in the lysate lanes (1 and 6). The final antibody, anti-*B. burgdorferi,* raised against borrelial whole cell lysate, was hence utilized here as a control for the experiment, since it should detect undetermined proteins from BbOMVs. As expected, the lysate bands exhibited strong signals throughout the lanes (Figure 3H, lanes 1 and 6). BbOMV lanes, on the other hand, illustrated smaller proteins, with bands at approximately the same locations as OspA, OspC, and p39, mentioned above (Figure 3H, lanes 3–5), suggesting the validity of the previous labels. Two additional bands were located from the BbOMV lanes (3–5), indicating either labelling of fragmented proteins or further proteins unspecified here (Figure 3H).

### 3.3. BbOMVs Did Not Induce Cell Death in Human Cells

Flow cytometry was utilized in analyzing the cytotoxic effect of BbOMVs in chondrosarcoma (SW1353) and skin fibroblast (BJ) cells. The cells were treated with *B. burgdorferi* spirochetes, RBs, and BbOMVs for 72 h, and double stained with pan-caspase inhibitor peptide conjugated to a fluorochrome and a ketone group (FAM-VAD-FMK) and a DNA label (7-AAD). Untreated and staurosporine (1 µM) treated cells were controls for viability and death, respectively. The double staining produced quadrant sections by which four different cell populations could be located: viable, early apoptotic, late apoptotic/necrotic and dead cells.

**Figure 4:**
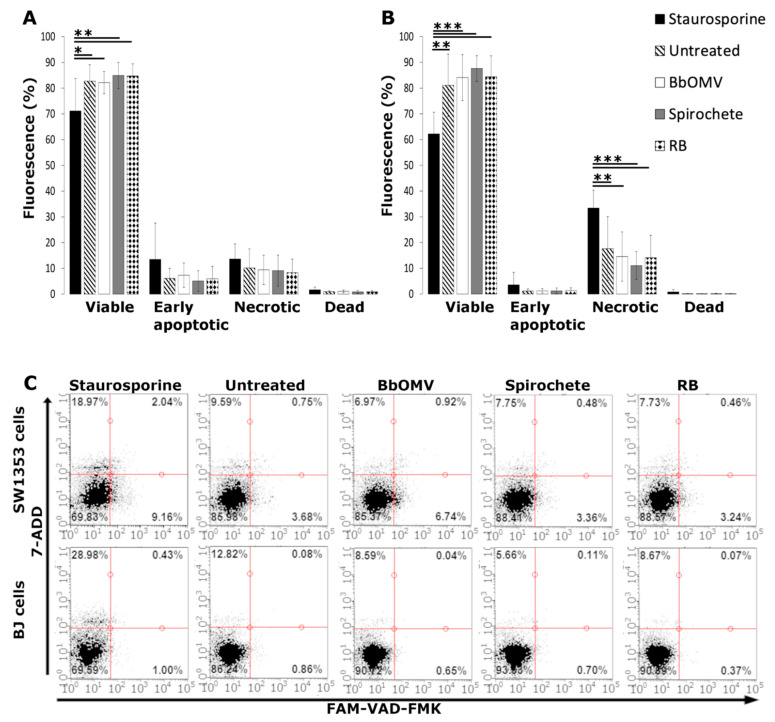
BbOMVs did not induce cell death in human cells. In order to detect different stages of cellular viability after infection, SW1353 (**A**) and BJ (**B**) cells were infected with *B. burgdorferi* spirochetes (MOI 40), RBs (MOI 40) and BbOMVs (50 µg/mL), respectively, for 72 h and double labelled with DNA (7-AAD) and pan-caspase inhibitor-fluorochrome complex (FAM-VAD-FMK) markers. Untreated cells were used as negative control and 1 µM staurosporine treated (2 h) cells were a positive control for apoptosis (cell death). The samples were analyzed with Guava easyCyte 8HT flow cytometer. Standard deviations from triplicate experiments. Statistical significance was compared to both negative and positive controls. * *p* ≤ 0.05; ** *p* ≤ 0.01; *** *p* ≤ 0.001. (**C**) Representative dot plots of SW1353 (top row) and BJ (bottom row) cells from the abovementioned experiment. In the graphs, viable cells are located at the bottom left corner, early apoptotic at the bottom right corner, late apoptotic/ necrotic cells at the top right corner, and dead cells at the top left corner.

The results demonstrated that neither *B. burgdorferi* spirochetes, nor RBs, nor BbOMVs induced apoptosis in the human cells, since both cell lines, SW1353 and BJ, had over 80% viability after 72 h (Figure 4A,B, respectively). Specifically, in the viable cell population of the SW1353 cells a significant difference (*p* ≤ 0.05) was observed between the staurosporine treated cells and the untreated and BbOMV coculture samples (Figure 4A). Moreover, a higher significant difference was detected between staurosporine and *B. burgdorferi* spirochete and RB samples (*p* ≤ 0.01) (Figure 4A). In the SW1353 cells, less than 10% of both the untreated and *Borrelia* cocultured samples had early apoptotic or late apoptotic/necrotic cells, respectively, after 72 h (Figure 4A). Furthermore, there were very few dead cells in each sample of SW1353 cells, thus further exhibiting the viability of SW1353 cells even after 72 h co-incubation with *B. burgdorferi* spirochetes, RBs, or BbOMVs. 

Similarly, in BJ cells there was minimal amount of early apoptotic (<2%) or dead (<1%) cells in both the untreated and each *Borrelia* cocultured samples (Figure 4B). However, significant differences were observed in both the viable and necrotic cell population of BJ cells. Specifically in the viable BJ cell population a significant difference between the staurosporine treated and untreated (*p* ≤ 0.01), and each *Borrelia* cocultured and staurosporine treated sample (*p* ≤ 0.001) were observed (Figure 4B). In the necrotic cell population significant differences were analyzed between the staurosporine and the untreated and BbOMV samples (*p* ≤ 0.01), as well as, between the staurosporine and *B. burgdorferi* spirochete and RB samples (*p* ≤ 0.001) (Figure 4B). Nevertheless, the untreated control exemplified more necrotic cells than *B. burgdorferi* spirochete, RB, or BbOMV cocultured samples, thus, further demonstrating the non-lethal effect of *B. burgdorferi* on BJ cells.

In Figure 4C, a representative dot plot of flow cytometry analysis of SW1353 (top) and BJ (bottom) staurosporine treated, untreated and *B. burgdorferi* BbOMVs, spirochete and RB cocultured samples can be located. In the quadrant plots the viable cell population is in the bottom left corner; the early apoptotic in the bottom right corner; the late apoptotic/ necrotic in the top right corner, and the dead cells in the top left corner. As can be seen from the plots of each sample, the majority of the cell populations are in the viable, bottom left corner, in both human cell lines (Figure 4C). 

## 4. Discussion

LB can progress into a debilitating and prolonged multisystemic disorder. Currently, it remains unclear how a *B. burgdorferi* infection can, at times, lead to such a sustained state of distress in patients. *Borrelia* is a pleomorphic bacterium with known persister morphologies such as round bodies and biofilms, which might contribute to the persistence of the disease [42,43,44]. However, the role of borrelial membrane vesicles in the pathogenicity of the disease has not been fully considered. After all, both whole bacterial cells, as well as borrelial antigens, have been detected in human and animal samples after antibiotic treatment [7,8,9,10]. The mechanism by which these antigens remain in tissues is not clear, but OMVs could potentially provide both a convenient hiding place and transport system for such persistent antigens. This study examined BbOMVs, first by characterizing them, and later by analyzing the cytotoxic consequences of these vesicles on human cells.

Bacterial OMVs vary in sizes. For instance, enterotoxigenic *Escherichia coli* has been shown to produce extracellular vesicles as large as 300 nm in diameter [45], while *Neisseria gonorrhoeae* produced membrane vesicles as tiny as 6 nm in diameter [23]. In a previous study, the researchers chemically induced borrelial membrane blebbing with citrate buffer and reported the formed BbOMVs to be 300–1000 nm in diameter [25]. Contrarily, we analyzed a total of 600 naturally formed BbOMVs from negatively stained EM micrographs and measured the diameters to range from 11 nm to ~108 nm, with an average of 33 nm (Figure 1). To the best of our knowledge, this is the first time the size of naturally occurring BbOMVs has been reported. Hence, naturally blebbed BbOMVs were 10–30 times smaller on average, than the chemically produced vesicles [25]. 

Analysis of TEM images demonstrated heterogeneously spherical vesicles with single bilayer membranes with both light and dense contents, thus confirming the purified material as BbOMVs (Figure 2A). Previous work on *Pseudomonas aeruginosa* demonstrated that empty membrane vesicles can take up extracellular DNA [46], further validating the role of extracellular vesicles as an instrument of communication for both the “sender” and the “receiver” of the vesicle. Hence, it was considered that some of the lighter BbOMVs seen here (Figure 2A) might be empty inside, and that *Borrelia* might utilize its vesicles in a similar manner as *Pseudomonas aeruginosa*. Therefore, BbOMVs might participate in LB-related autoimmune disorders by intaking host extracellular DNA and fooling the host immune system into perceiving it as foreign, thus instigating the host to attack itself. However, further validation for this hypothesis is required.

Bacterial membrane vesicles have been identified to carry different types of cargoes [11,12,47]. In Table 1, some of the characteristics of BbOMVs identified thus far can be located. Here, further characterization of BbOMVs confirmed the presence of nucleotides in the form of double stranded DNA (Figure 3A), as has been previously described [29,38]. Furthermore, Malge and colleagues have identified plasmid-encoded RNA transcripts to be enriched in BbOMVs [30]. The containment of genetic information inside BbOMVs would suggest the transfer of information between bacterial cells, if not also, between the bacterium and the host. 

Previous analyses have identified several known antigenic lipoproteins, such as OspA, OspB, OspC, and OspD, contained in BbOMVs [16,24,26,28]. Similar to previous findings [16,24,26,28], we confirmed the expression of OspA and OspC in BbOMVs (Figure 3D,E). OspA is expressed while the spirochete is in the tick gut, but both temperature and tick feeding trigger a change to OspC expression [48]. Hence, *Borrelia* utilizes OspC during transmission from the tick vector to the mammalian host, and the lipoprotein is required for establishing an infection [49]. Furthermore, a recent study established that OspC has antiphagocytic properties, thus promoting borrelial immune evasion during early dissemination [50]. Both OspA and OspC have been demonstrated to be immunogenic in patients with arthritic symptoms, with increased IgG response to OspA during later stages (months to years) of Lyme arthritis [51]. Therefore, the presence of both OspA and OspC in BbOMVs could affect the pathology of LB by inducing an inflammatory response in the host.

Curiously, OspC bands were barely visible in the bacterial whole cell lysate controls (lanes 1 and 6 in Figure 3E). Enterotoxin, from enterotoxigenic *Escherichia coli,* and aminopeptidase, from *Pseudomonas aeruginosa,* have been demonstrated to be enriched in the OMVs of these bacteria [52,53]. Both proteins, enterotoxin and aminopeptidase, were shown to increase vesicle association with endothelial cells [52,53]. Thus, the immunoblot results examined here might suggest a similar occurrence for OspC in *B. burgdorferi*, since OspC is necessary for borrelial infectivity [49]. Moreover, as OspC can protect the bacterial cell from phagocytosis [50], it could be possible for the bacterium to disseminate OspC in advance to establish a suitable environment for the bacterium to initiate infection and survive the host immune response.

We further analyzed, and detected, immunogenic proteins peptidoglycan and p39 (basic membrane protein A) in the vesicles (Figure 3F,G, respectively). Both p39 and borrelial peptidoglycan have been identified as markers for Lyme arthritis [7,54]. Since Lyme arthritis is a late manifestation of *B. burgdorferi* infection [55], both peptidoglycan and p39 would have to remain long-term in the host. BbOMVs would provide a convenient mechanism for these antigenic markers to persist in the host during the course of LB, and to survive antibiotic treatment.

Bacterial OMVs can induce immunological consequences in the host. *Helicobacter pylori*, for instance, has been observed to induce growth arrest, increased cytotoxicity, and IL-8 production in gastric epithelial cells through OMV production [56]. *E. coli*, on the other hand, has been shown to secrete Shiga toxins via its OMVs [57]. In one study, bacterial lipopeptides were found to induce neuronal dysfunction both in mice and cultured neurons, suggesting a pathogenic role for lipoproteins without the presence of live bacteria [58]. As the investigated BbOMVs contained lipoproteins OspA, OspC, and p39 (Figure 3D,E,G), some of the neurological symptoms in patients seen post-treatment could be the result of BbOMV-induced pathology.

BbOMVs contain immunogenic antigens and can adhere to endothelial cells; however, the vesicles do not induce cell death in these cells [24]. Similarly, in our study, BbOMVs cocultured with human chondrosarcoma (SW1353) and skin fibroblast (BJ) cells did not increase cell death as compared to the staurosporine treated controls (Figure 4). Furthermore, corresponding to our previous findings [41], neither spirochetes nor RBs induced cell death in these two human cell lines. Hence, as none of the *B. burgdorferi* samples, spirochetes, RBs, or BbOMVs induced death in these two human cell lines, it would seem that *Borrelia* is not cytotoxic to chondrosarcoma or dermal fibroblast cells.

Currently, the persistence of *Borrelia* or borrelial antigens in LB patients requires further examination and validation. Moreover, the role of BbOMVs in the pathology of LB should be investigated to a greater extent. However, due to the presence of known immunogenic markers OspA, OspC, peptidoglycan, and p39, examined here, it could be suggested that BbOMVs hold the potential for inducing an immune response in the host. Furthermore, it has been suggested that BbOMVs could participate in the induction of autoimmune-related consequences in patients via membrane lipid exchange with host cells [59]. Moreover, as bacterial OMVs have been demonstrated to be capable of intaking extracellular DNA [46], BbOMVs could function in a similar manner. Therefore, BbOMVs possess several mechanisms for aiding *Borrelia* in both initiating an infection, as well as sustaining a prolonged immune response, and possibly even initiating an autoimmune response via self-antigens in the form of host lipids and/or DNA.

## 5. Conclusions

We examined the characteristics of borrelial outer membrane vesicles, and the possible cytotoxic consequences induced by these vesicles in human cells. For the first time, the size of naturally formed BbOMVs was reported, as well as the lack of cell death in chondrosarcoma and dermal fibroblast cells. We demonstrated that BbOMVs contained known antigenic markers OspA, OspC, peptidoglycan, and p39. We propose that *Borrelia* could utilize BbOMVs as a decoy for the host immune system by disseminating immunogenic proteins such as OspA, OspC, peptidoglycan, and p39, thus avoiding detection and elimination itself. Moreover, BbOMVs could be utilized by the bacterium as persistent antigens, consequently sustaining a prolonged immune response by the host. Hence, the pathogenic role of BbOMVs should be recognized and further examined.

## Figures and Tables

**Table 1:** Known characteristics of *B. burgdorferi* outer membrane vesicles.

Marker	Molecular Mass (kDa)	Reference
OspA	29/31	[16,24,25,26,28]
OspB	32/34	[16,24,25,26,28]
OspC	18	[28]
OspD	28/29	[24,25,28]
Lp6.6/La7/p66	8/22/68	[28]
p13	19	[28,32]
p39 (BmpA)	37	[28,32]
Lack of flagella	37.5/41	[16,24,26]
Other unidentified proteins	110/50	[27]
	64/30/28/21/19/15/14	[16]
	23	[26]
	19.5	[25]
Enolase	47	[32]
BSA	66	[27]
**Nucleotide form and Origin**		
DNA	Linear/circular	[23]
	Linear/circular plasmids	[29]
	Linear chromosomal	[29]
RNA transcripts	Mostly from plasmids	[30]
**Other Markers**		
Porins	0.6/12–13 nS	[25]
Diameter (chemically produced)	300–1000 nm	[25]

## Data Availability

Data is contained within the article or Supplementary Material.

## Supplementary Materials

The following supporting information can be downloaded at: https://www.mdpi.com/article/10.3390/microorganisms10020212/s1, Figure S1: Only 1.8% of residual borrelial particles were located from BbOMV purification.
