# Supplementary material for: Borrelia burgdorferi Outer Membrane Vesicles Contain Antigenic Proteins, but Do Not Induce Cell Death in Human Cells
Source: Microorganisms. 2022 Jan 19;10(2):212. doi: 10.3390/microorganisms10020212 (PMC8878412; doi:10.3390/microorganisms10020212)
Supplement: Supplementary file 1 [file microorganisms-10-00212-s001.zip › microorganisms-1531798-supplementary.pdf]

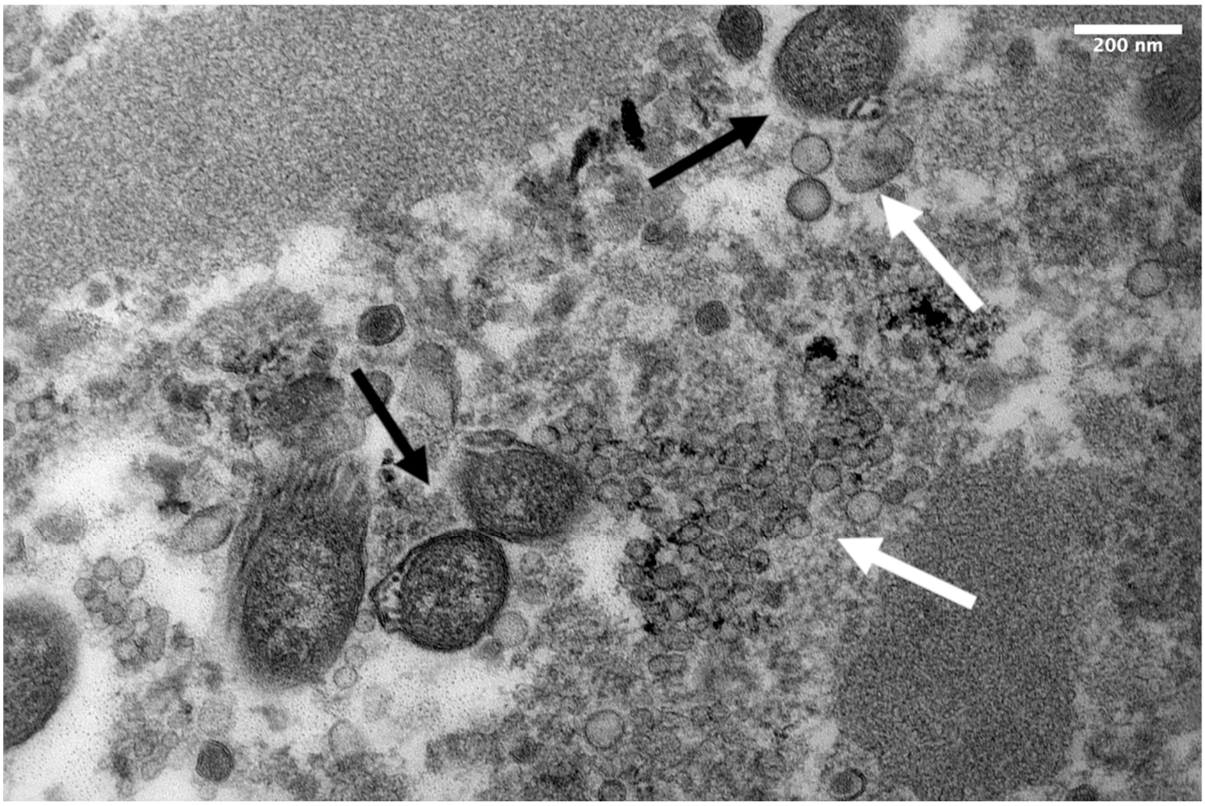

Figure S1. Only 1.8% of residual *Borrelia* were located from BbOMV purification. Transmission electron micrographs of epon embedded samples of purified BbOMVs. Due to the discovery of residual *B. burgdorferi* spirochetes in the purified BbOMV sample, a calculation for the ratio of residual *Borrelia* and BbOMVs was performed. From 30 randomly selected micrographs (16-bit) with 5.04 mm^2 areas, the ratio of BbOMVs and residual *Borrelia* were analyzed. In total only 1.8% of *Borrelia* spirochetes was uncovered. Black arrows point to bacterial cells found mixed in with the vesicles (white arrows).
